# A Vicious Cycle: A Cross-Sectional Study of Canine Tail-Chasing and Human Responses to It, Using a Free Video-Sharing Website

**DOI:** 10.1371/journal.pone.0026553

**Published:** 2011-11-09

**Authors:** Charlotte C. Burn

**Affiliations:** Veterinary Clinical Sciences, The Royal Veterinary College, North Mymms, Hertfordshire, United Kingdom; Umeå University, Sweden

## Abstract

Tail-chasing is widely celebrated as normal canine behaviour in cultural references. However, all previous scientific studies of tail-chasing or ‘spinning’ have comprised small clinical populations of dogs with neurological, compulsive or other pathological conditions; most were ultimately euthanased. Thus, there is great disparity between scientific and public information on tail-chasing. I gathered data on the first large (*n* = 400), non-clinical tail-chasing population, made possible through a vast, free, online video repository, YouTube™. The demographics of this online population are described and discussed. Approximately one third of tail-chasing dogs showed clinical signs, including habitual (daily or ‘all the time’) or perseverative (difficult to distract) performance of the behaviour. These signs were observed across diverse breeds. Clinical signs appeared virtually unrecognised by the video owners and commenting viewers; laughter was recorded in 55% of videos, encouragement in 43%, and the commonest viewer descriptors were that the behaviour was ‘funny’ (46%) or ‘cute’ (42%). Habitual tail-chasers had 6.5+/−2.3 times the odds of being described as ‘Stupid’ than other dogs, and perseverative dogs were 6.8+/−2.1 times more frequently described as ‘Funny’ than distractible ones were. Compared with breed- and age-matched control videos, tail-chasing videos were significantly more often indoors and with a computer/television screen switched on. These findings highlight that tail-chasing is sometimes pathological, but can remain untreated, or even be encouraged, because of an assumption that it is ‘normal’ dog behaviour. The enormous viewing figures that YouTube™ attracts (mean+/−s.e. = 863+/−197 viewings per tail-chasing video) suggest that this perception will be further reinforced, without effective intervention.

## Introduction

Tail-chasing in dogs is widely celebrated in cultural references, such as its depiction in the cheerful, repetitive phrases of Chopin's Minute Waltz [1], and as performed by Sirius Black's *animagus* dog, Padfoot, in the Harry Potter series, when it is accompanied by a ‘joyful bark’ [2]. However, scientific literature exclusively refers to tail-chasing – or ‘spinning’, when the behaviour is not necessarily focussed towards the tail – in clinical contexts, because it can indicate welfare problems of varying severity, e.g. [3], [4], [5]. The most common reported diagnosis is canine compulsive disorder [6], [7], but other conditions, such as dermatitis or anal sacculitis [8], are also reported. Even in otherwise healthy dogs, the behaviour could indicate externally triggered welfare problems including lack of stimulation (‘boredom’), insufficient exercise, or various stressful situations [4], [7], [9]. Nevertheless, tail-chasing can simply comprise play or exercise in many dogs, and these ‘normal’ tail-chasers have never yet been included in scientific publications, partly because the sporadic nature of the behaviour makes it difficult to study.

Clinical texts, e.g. [3], [4], [10], [11], often propose that compulsive tail-chasing develops from repeated exposure to triggering events or situations, but the behaviour gradually becomes dissociated from the original trigger, occurring ever more frequently in increasingly diverse contexts. In other words, the behaviour might develop through a vicious cycle. Like many stereotypic behaviours, tail-chasing can sometimes be temporarily eliminated by the opioid blocker, naloxone [12]. Attempted treatments for compulsive tail-chasing include behavioural therapy alongside drugs, including the tricyclic antidepressant, clomipramine, the selective serotonin reuptake inhibitor, fluoxetine [6], [9], and the NMDA receptor blocker, memantine [7]. Tail-amputation has no reported success, and the problem can be so intractable, and distressing for the owners, that dogs are euthanased [7], [12]. Indeed, all 32 dogs in Blackshaw et al.'s [12] study – the largest study to date – were euthanased due to the persistence of their condition.

Several breeds are prone to compulsive tail-chasing, including Bull Terriers [12], German Shepherds [6] and Anatolian sheepdogs [9]. However, the sample sizes of clinical studies to date have been too small to rule out high propensities in other breeds too, such as Jack Russells and West Highland White Terriers [12]. Breed differences could arise from environmental (e.g. opportunities to exercise) and/or genetic factors. If the latter, the behaviour could have been artificially selected for, even indirectly if tail-chasing is linked with a desirable characteristic, as with many inherited defects [13].

**Figure 1:**
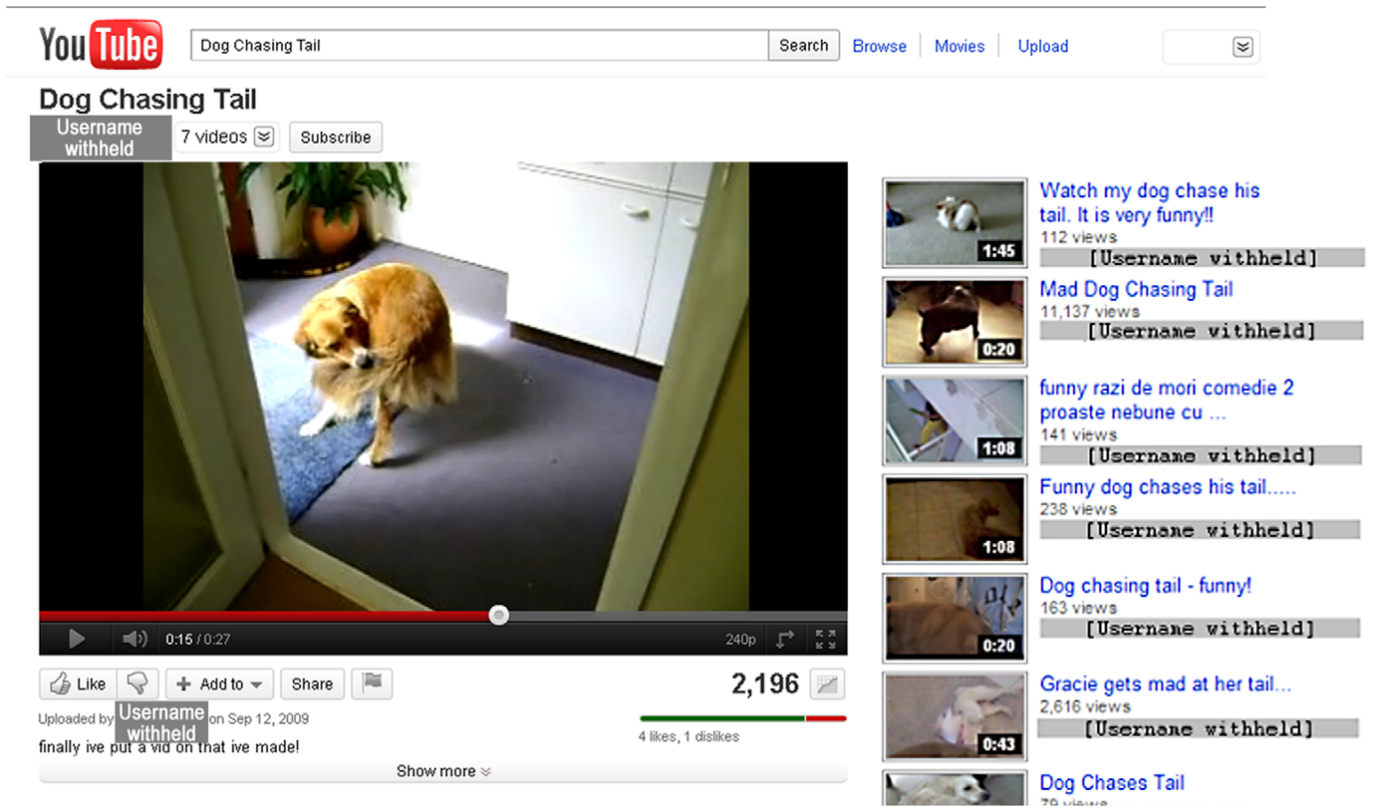
Screenshot of a video of a Golden Retriever chasing its tail on YouTube™. The sidebar on the right also offers views links to related videos, showing a thumbnail of the video content, the video title, and the number of times the video has been viewed. The usernames are withheld here for privacy reasons, but on YouTube™ they are hyperlinked to the uploaders' homepages, which usually contain information about their age, sex, country, and their other videos.

Despite the general renown of the behaviour and its potential severity in clinical cases, little is known about tail-chasing in home contexts or when no clinical causes have been diagnosed. Yet, a search for “dog chasing tail” on the most popular video-sharing website [14], YouTube™, returned almost 3500 hits in 2010. These videos provide a new opportunity for a hitherto untapped insight into tail-chasing in non-clinical contexts, and will include many ‘normal’ dogs (those with no relevant clinical diagnosis). For the first time, a large sample size is rapidly available and economically feasible. Furthermore, the videos reveal environments and contexts in which tail-chasing occurs, often together with audible and written responses of human observers (Figure 1).

Despite the increasing accessibility of broadband and video cameras/phones to a wide demographic, the dogs and humans on YouTube™ will not represent *all* dogs and humans; indeed truly representative sampling eludes most population studies. Dogs that tail-chase very rarely are likely to be under-represented, as videographers would have to catch the behaviour at exactly the right place and time. Conversely, dogs with clinical diagnoses may also be under-represented if owners are embarrassed (but not if they wish to raise awareness). Thus, the tail-chasing dogs on YouTube™ should approximately represent the centre of the normal distribution of dogs that chase their tails at some point in their lives. As with other survey methods, the use of video-sharing websites requires similar caution in generalizing conclusions beyond the sample population, because the populations are usually non-random and self-selecting to some extent. However, data from video-sharing websites reflects directly observed behaviour (rather than relying on respondents' descriptions), and data are unprompted by the researcher, so they are less likely to be biased towards the study purposes.

To date, video-sharing websites, such as YouTube™, have been studied regarding their potential for disseminating information to the public, in contexts including tobacco use [15], immunization [16] and sunbed use [17]. More recently, the actual video content has begun to be explored epidemiologically, providing insight into an asphyxiation ‘game’ in teenagers (using 65 video clips) [18], and into dietary messages given by adults to children playing with toy kitchens (115 clips) [19]. The current study goes further, using a larger sample size, plus a control group to examine the characteristics of and responses to tail-chasing in domestic dogs.

My aims were to describe (i) canine breed/morphological and (ii) behavioural characteristics, and the (iii) animal welfare implications and (iv) broad environmental contexts, associated with tail-chasing; and also (v) to describe human responses to it on YouTube™. I made no clinical diagnoses from the videos, but could broadly infer certain animal welfare implications from visible injuries and characteristics commonly associated with perseverative abnormal behaviours, including both frequent performance and persistence in the face of distraction.

## Methods

### Description of tail-chasing videos

I identified tail-chasing videos using the search term “dog chasing tail” on YouTube, which returned 3340 hits in November 2009. The videos were continually but gradually shuffled by YouTube's confidential search algorithms. Between Nov 2009 and August 2010, I collected data from the first 400 videos of the returned hits, subject to the following exclusion criteria: only one video was used per ‘uploader’ (person who uploaded a video to their YouTube™ account); and very dark or pixelated videos, or those not showing a domestic dog tail-chasing or spinning were discarded; photographic collages, professional videos, and advertisements were excluded, and in video collages, only the first continuous shot was used. It is worth noting that in some cases, the uploader may neither have owned the dog, nor have taken the footage themselves.

The following details were recorded from the videos (further details in Table S1):

- Clip ID and URL
- the reported sex, age and nationality of the uploader
- dog breed, sex and age
- dog tail morphology
- relevant human and dog behaviour observed in the video (summarized in Table 1)
- environmental context (indoors or outdoors; television switched on, off or unknown)
- relevant descriptive comments by the uploader and viewers (summarised in Table 2).

**Table 1:** Brief description of behavioural data collected from YouTube, and associations between them.

Behavioural characteristic	Description	Proportion of videos showing the characteristic (excluding videos with missing values)	Significant associations (↑ = positive association; ↓ = negative association	Odds ratio +/− S.E.; DF; P-value
Tail-chasing frequency as indicated by uploader comments*	‘Habitual’ (e.g. daily, “all the time”, “a lot”, “spends hours” tail-chasing, the dog is “obsessed”);‘Periodic’ (e.g. “from time to time”, “regularly”, “[the dog] usually tail-chases when…”);or ‘Rare’ (e.g. “[the dog] rarely does this”, I “managed to catch” the dog tail-chasing)	Habitual: 26/86 (30.2%);Periodic: 49/86 (57.0%);Rare: 11/86 (12.8%)	↑Difficult to distract	8.06+/−2.50; 9; 0.049
“	“	“	↑ ‘Stupid’ in uploader comments	6.52+/−2.33; 23; 0.037
Difficult to distract	The dog did not stop chasing for more than 5 s despite a potential distraction (e.g. the owner commanded the dog to do something other than tail-chase, a sudden noise, or the dog collided with something hard enough to impede its progress)	76/198 (38.4%)	↓Play	0.16+/−1.70; 102; 0.001
“	“	“	↓Encouragement	0.28+/−1.40; 102; 0.000
“	“	“	↑ ‘Funny’ in public comments	6.82+/−2.09; 24; 0.016
“	“	“	Also see Habitual tail-chasing frequency	-
Vocalisations heard during or within 5 s of tail-chasing	Barking	54/366 (14.8%)	↓Television and computer use	0.30+/−1.51; 201; 0.004
“	“	“	↑Tail wagging	2.30+/−1.45; 201; 0.026
“	Growling	75/353 (21.2%)	↑Hunter Group (Parker et al., 2007)	2.66+/−1.63; 83; 0.050
“	“	“	↑Age (i.e. adults)	2.30+/−1.40; 206; 0.013
“	Whining	4/354 (1.1%)	(too rare to test)	-
Collision	Dog collided with an object during or up to 30 s after tail-chasing	101/393 (25.7%)	↓Play	0.37+/−1.53; 262; 0.019
“	“	“	↑Laughter	2.12+/−1.32; 230; 0.007
Play behaviour	Within 5 s of a chasing bout, the dog exhibits a play bow (characteristic posture with the forelegs extended on the ground), object play (manipulation of a toy or other available object), social play (with human or conspecific), or locomotor play (e.g., bounding, rolling)	66/389 (17.0%)	↑Tail wagging	3.89+/−1.40; 259; 0.000
“	“	“	↓Age	0.24+/−1.39; 259; 0.000
“	“	“	↑Outside	3.26+/−1.63; 260; 0.016
“	“	“	↓Funny	0.04+/−3.60; 68; 0.023
“	“	“	Also see Difficult to distract, and Collisions	-
Tail wagging	Dog rhythmically moves its tail laterally at least twice in each direction within 5 s of a chasing bout, rather than it remaining inanimate or moving irregularly	135/393 (25.7%)	↑Age	2.77+/−1.36; 207; 0.001
“	“	“	↑Television and computer use	2.15+/−1.33; 237; 0.008
“	“	“	↑Mastiff-terriers	2.67+/−1.63; 84; 0.046
“	“	“	Also see Play Behaviour and Barking	-
Mouths tail	Dog is clearly seen to bite, lick or hold the tail or hindquarters/hind leg in its mouth for at least 1 s	248/392 (63.3%)	↑Laughter	1.78+/−1.27; 235; 0.018
“	“	“	↑‘Stupid’ in uploader comments	4.16+/−1.67; 154; 0.006

When videos had no sound-track or the soundtrack was replaced by music, missing values were recorded for data reliant on sound; similarly missing values were recorded for videos without relevant comments or where the behaviour could not be clearly seen. The proportion of tail-chasing videos (excluding those with missing values) showing each characteristic is displayed, along with any significant associations with relevant predictors, for which the odds ratios, degrees of freedom, and P-values are displayed. *This odds ratio was calculated from a model using ‘Habitual’ vs other frequencies as a binary variable.

**Table 2:** Human encouragement and responses to tail-chasing in dogs on YouTube™.

Human response to tail-chasing (*n* = number of valid videos)	Proportion of videos (excluding videos with missing values)	Examples or synonyms (where relevant)
*Human behaviour*	-	-
Laughter	199/362 (55.0%)	Female: 66.4%; male 18.6%; both sexes: 15.0%
Verbal encouragement	119/362 (32.9%)	“Get your tail!”, “Get it!”
‘Growling’ at dog	6/321 (1.9%)	
Physical manipulation	74/371 (19.9%)	Placing the tail in the mouth, pulling or pinching the tail, waving the tail near the dog's face, pushing the hindquarters
Tail attachment	14/371 (3.8%)	Attaching hair bands, dog toys or treats, a bottle, a section of plastic piping, or string to the tail
Verbal praise	12/362 (3.3%)	“Good dog”, “Good girl/boy”, and other variants
Physical praise	2/371 (0.6%)	Patting or stroking the dog, or feeding it a treat, after a chasing bout
*Uploader description*	-	-
‘Funny’	149/253 (58.9%)	“Funny”, “haha”, “lol” (laugh out loud), “hilarious”, “comedy”, “humour”, “XD” (a laughing emoticon), “lmao” (laugh my ass off)
‘Crazy’	65/250 (26.0%)	“Crazy”, “mad” (but not “gets mad” or “mad at” as these indicate perceived anger), “insane”, “mental”, “maniac”, “nuts”, “psycho”, “nutcase”
‘Cute’	47/250 (18.8%)	“Cute”, “cutie”, “sweet”, “aww”, “adorable”
‘Stupid’	38/251 (15.1%)	“Stupid”, “retard/retarded”, “nerd”, “dumb”, “duh/doh”, “dumbass”, “dopey”, “idiot”, “moron”
‘Silly’	28/250 (11.2%)	“Silly”, “Goofy”
‘Fun’	19/250 (7.6%)	“Fun”, “amusing”, “entertainment”
‘Play’	12/250 (4.8%)	“Play”, “playing”, “game”, “playful”
‘Dizzy’	11/250 (4.4%)	“Dizzy”
‘Weird’	10/250 (4.0%)	“Weird”
‘Tricks’	8/249 (3.2%)	Tail-chasing is the dog's “party trick”
‘Awesome’	8/250 (3.2%)	“Awesome”, “cool”, “amazing”, “wow”
‘Bored’	5/250 (2.0%)	“Bored”
‘Hyper’	4/250 (1.6%)	“Hyper”, “hyperactive”, “energetic”
Other	N/A	Angry, classic, clever, confused, crack up, curious, dirty, enjoy, freak, frenzy, frustrated, inner battle, itchy, loser, nerd, nice, obsessed (x 2), possessed, serious problems, smart, spaz, tipsy, torture, wild, wrong, “I love that my dog actually chases her tail”
Explanations given	N/A	[The dog…] “loves/likes to tail-chase” (x6), “hates his tail”, is “entertaining herself”, is “having fun”, is “either bored or has high cholesterol”, “enjoys the dizziness”, does it “out of dominance”, “puts on a little show”, “needs prozac”, “chases on command” (x2), is “still a puppy”, “hasn't figured [his tail] is connected to him”, is showing “typical dog behaviour”, is playing “his favourite game”
*Viewer comments*	-	-
‘Funny’	64/138 (46.0%)	As for ‘Uploader description’, plus “hilarious”
‘Cute’	58/138 (41.7%)	As for ‘Uploader description’
‘Awesome’	16/138 (11.5%)	As for ‘Uploader description’, plus “impressive”
‘Stupid’	11/138 (7.9%)	As for ‘Uploader description’, plus “daft”, “not that smart”
‘Crazy’	4/138 (3.6%)	As for ‘Uploader description’, plus “bonkers”
Other	N/A	“Great” (x2), “excellent”, “nice” (x3), “priceless”, “entertaining”, “weird”, “gay”, “fun” (x2), “cruel”, “animal abuse”, “I wonder why they do that”, “My dog does/did that too” (x7), “My dog bites his tail to the point of bleeding”, “My dog spins/chases faster than yours” (x4), “Dog chasing tail never gets old”, “I want your dog”, “I've never seen a dog do that”, “I feel bad for him”, “repetitive behaviours need to be checked by a vet”, “I love it when dogs and cats do that”
Explanations given	N/A	[The dog…] has “high cholesterol” (x2), has “canine compulsive disorder”, is in “pain/discomfort”, has “Schizophrenia”, needs “the doggie chiropractor”, is “happy”, needs “toys”, “doesn't know [the tail] is part of their body yet”, has an “itchy tail”, has “worms”, is “hyper”, is “bored”, is “showing off”, has “a flea stuck in his tail”

The percentages of videos are arranged in order of magnitude for each general category. The words that were accepted as valid synonyms for comment categories were shown. These were accepted only if they were consistent within the context of the whole comment, e.g. a comment was not included in the counts for ‘funny’ if the comment actually stated that the video was ‘not funny’, even though the keyword was present in the comment.

I structurally defined all the behaviours scored according to an ethogram (Table S1 and S2), and systematically categorized human comments after data collection using defined criteria (Table 2).

### Comparisons of tail morphology and environmental context in breed-matched controls

I compared tail-chasing videos against 400 breed-matched control (non-tail-chasing) videos, to investigate associations between tail-chasing and tail morphology, such as whether docked tails were more or less frequently seen in tail-chasing versus control videos. The control videos were also used to identify whether dogs were more frequently indoors, and whether a television, computer, radio or music was switched on when tail-chasing. Breed- and age-matching was important because these factors affect the likelihood that dogs are taken outdoors and that their tails are docked. My control search terms were “[dog breed name]”+“dog” or “puppy” as appropriate to match each tail-chasing video. The first control video not yet scored for that breed was used in each case. Exclusion criteria were as before, but additionally, videos were excluded if the tail could not be clearly seen; if the control video included tail-chasing or spinning; or if the video seemed to involve animal cruelty, for ethical reasons (e.g. dog fights). The ensuing control videos included diverse footage: for example, dogs playing, vocalising, performing ‘tricks’, eating, dreaming, exercising, exploring novel stimuli, or interacting with other dogs, other pets, or humans.

### Observer reliability

A subset of the variables described in Table S2 & S3, encompassing the more subjective aspects of dog and human behaviour, were checked for inter- and intra-observer reliability using 10% of the tail-chasing videos. Kappa observer reliability statistics are meaningless in overly homogenous samples [20]–[22], so Hoehler [21] suggests that investigators should ‘concentrate on obtaining populations with trait prevalence near 50% rather than searching for statistical methods to rescue inefficient experiments.” The 40 videos were therefore selected (using my ratings as the primary observer) to optimize the prevalence index for as many variables as possible, avoiding overly homogenous samples and allowing even rare scores to be tested [20], [21]. For example, only 46 videos had comments revealing the dog's tail-chasing frequency as well as having a potentially distracting event occurring during the video, so 35 of these videos were included in the reliability sample (representing habitual, periodic and rare tail-chasing, in both perseverative (difficult to distract) and non-perseverative dogs). This meant that for key variables, such as tail-chasing frequency, distractibility, or play behaviour, the prevalence index was <0.4 [20], so no variable was too rare to test.

The order in which videos were re-watched was randomized. The other observer (OHB; see Acknowledgements) was an experienced observer of animal behaviour, and was blind to the hypotheses being tested. He received five practice videos for which he could see my original scores, and he was given a detailed description of the scoring criteria for each variable (Table S2), but he received no other training.

Intra- and inter-observer agreement was tested using Fleiss' Kappa statistics for binary variables, and Kendall's W for ordinal variables (Minitab 15). Thresholds for clinical acceptability were defined as Moderate (*κ* or *W*≥0.4), Substantial (≥0.6), or Excellent (≥0.8) according to convention, e.g. [22]. Only scores for panting behaviour failed to attain at least Moderate reliability, so results for that variable are not reported. The observer reliability scores are shown in Table S3.

### Statistical methods

Within the 400 tail-chasing videos, I tested associations between specific tail-chasing behaviours and their predictors (other behaviours, dog characteristics, and human responses) using generalized linear mixed models (glmmPQL and glmmML in R). I included breed as a random factor in every model to control for non-independence of similar dogs, and compared breed groups (defined according to both the UK Kennel Club and genetic groupings found by Parker et al. [23]) either as random or as fixed factors in alternative models. Breed was nested within breed group. Video-length was always included, because certain events (e.g. play behaviour or potential distractions) will have been more likely to be observed in longer videos. For analyses of clinically relevant predictors, dogs with objects attached to their tails were excluded, because their tail-chasing was not necessarily ever a self-initiated behaviour.

I also used generalized linear mixed models, as before, to compare tail-chasing and control videos. In these analyses, tail morphology, the in- or outdoor location, and television/computer/radio activity were used as predictors.

I selected models using Akaike information criteria, and identified (and thus avoided) multicollinearity using inflated standard error terms. The α-level for statistical significance was set at P≤0.05 in this exploratory study [24]; the number of independent tests for each dependent variable ranged from six to 16, depending on the hypotheses relating to that variable. Of the total 76 tests carried out, just under four (5%) of the seemingly significant results can therefore be expected to be Type I errors, but follow up studies will be required to reveal which results can and cannot be replicated. No correction for multiple testing has been done here, because the risk of Type II errors, failing to report potentially significant results, is considered more serious in exploratory studies than that of Type I errors [24].

## Results

### Uploader and video characteristics

Of the 400 uploaders of the tail-chasing videos, 69.0% were from the USA, 13.8% from the UK, 5.8% from Canada, and 9.8% from 19 other countries. There was no significant sex bias in uploaders: 30% were female, 24% male and 46% undeclared (Binomial test of 119 females of the 215 declared: *P* = 0.133). The mean (s.e.) reported age of uploaders was 27.5+/−0.44, ranging between 11 and 68 years.

The mean tail-chasing video length was 59.8+/−2.8 s. Each video had a mean of 863+/−197 viewings by May 2011 (maximum = 58,613), giving a cumulative viewing figure of 313,225 for the 400 videos included here.

### Tail-chasing characteristics and their associations

Associations between dog behaviour characteristics and context (excluding dogs with objects attached to their tails) are shown in Table 1. Of the 86 tail-chasing videos that had comments describing the frequency of tail-chasing, about 30% of dogs were stated as chasing their tails habitually (e.g. daily or ‘all the time’, rather than ‘periodically’ or ‘rarely’ (Table 1; Table S1), which is a clinical criterion for classifying tail-chasing as compulsive [7], [25]).

Approximately 38% of dogs appeared difficult to distract, or ‘perseverative’ during tail-chasing. Perseverative dogs were more likely to tail-chase habitually and to collide with objects when tail-chasing, and they were less likely to show play behaviours than were other tail-chasing dogs (Table 1). Hair-loss from the tail or hind-quarters was seen in 1.25% of the tail-chasing dogs and there were no comments that suggested uploaders or viewers considered this as an indication of the tail-chasing being a potential clinical problem.

Play behaviours (defined in Table 1) were interspersed with tail-chasing bouts in 17% of videos, and were more likely to be seen in puppies than older dogs. When indoors, tail-chasing was less likely to include play behaviour than when outdoors, and with a screen switched on, tail-chasing dogs were less likely to bark but more likely to wag their tails (Table 1).

Problematic tail-chasing (as indicated by the percentage of all tail-chasing videos that appeared perseverative or habitual per breed group) was distributed widely across diverse Kennel Club breed groups (Table 3). The highest proportion of perseverative tail-chasing was observed in toy breeds (56% of videos), followed by crossbreeds (43%) and terriers and working dogs (42% of both), but around one quarter of videos of gundogs, hounds, and utility breeds also showed evidence for perseveration. Few breed groups contained enough videos to enable assessment of tail-chasing frequency, but of those with at least 10 such clips, the highest proportion of habitual tail-chasing was observed in crossbreeds (52%) and terriers (38%). The five dogs with visible hair-loss or injury to the tail or hindquarters comprised two German Shepherds, one Labrador-Staffordshire Bull Terrier cross, one Labrador and one Parsons Jack Russell Terrier.

**Table 3:** Perseverative and habitual tail-chasing described by Kennel Club group.

Kennel Club Breed group	Total tail-chasing videos (*n*)	Perseveration				Tail-chasing frequency				
		Distractible (*n*)	Perseverative (*n*)	Percentage perseverative	Breeds exhibiting perseveration	Rare (*n*)	Periodic (*n*)	Habitual (*n*)	Percentage habitual	Breeds exhibiting habitual tail-chasing
Gundog	56	22	8	26.7	Goldendoodle, Golden Retriever, Labrador	2	9	3	21.4	Labrador, Springer Spaniel
Hound	21	9	3	25.0	Beagle, Dachshund	1	1	0	0.0	N/A
Pastoral	28	5	0	0.0	N/A	1	5	1	14.3	Shetland Sheepdog
Terrier	86	28	20	41.7	American Staffordshire Bull Terrier, Jack Russell Terrier, Patterdale Terrier, Pitbull, Staffordshire Bull Terrier, Yorkshire Terrier	3	7	6	37.5	American Staffordshire Bull Terrier, Jack Russell Terrier, Patterdale Terrier, Pitbull Terrier, Staffordshire Bull Terrier
Toy	56	11	14	56.0	Chihuahua, Havenese, Papillon, Pekingese, Pug	3	10	2	13.3	Chihuahua, Shih Tzu
Utility	29	10	3	23.1	Lhasa Apso, Shih Tzu	0	4	2	33.3	Lhasa Apso
Working dog	24	7	5	41.7	Bernese Mountain Dog, Boxer	1	2	0	0.0	N/A
Crossbreeds	100	30	23	43.4	N/A	0	11	12	52.2	N/A

Breeds are grouped according to the Kennel Club, which takes into account the breed history and general usage. They can also be grouped both genetically, as described by Parker et al. (2007), but those data are not shown here because not all recognised breeds have been genetically characterised according to that system to date. Representative breeds that showed perseverative or habitual tail-chasing are listed for each breed group; these were identified from uploader descriptions, or if no breed was stated, the breed was estimated from the appearance of the dog. Only those videos that included a potentially distracting event (*n* = 198) are included in the figures for perseveration, and only those with comments describing the tail-chasing frequency (*n* = 86) are included in the habitual chasing calculations.

### Human responses and descriptions of tail-chasing videos

While 69.3% of tail-chasing videos were categorized as ‘Pets and Animals’, 18.8% were categorized as ‘Comedy’ and 6.3% as ‘Entertainment’.

Human responses to tail-chasing are shown in Table 2. In 55% of videos, laughter could be heard, and this was significantly more likely to be female (in 81.6% of 114 clips with only one sex laughing; Binomial test: *P*<0.001). Laughter was positively associated with encouragement of the dog (Odds +/− S.E. = 2.83+/−1.28; DF = 234; *P*<0.001), but there were no significant associations with tail-chasing frequency or perseveration. Verbal or physical encouragement or praise was noted in 43% of videos, including attaching objects to the tail in almost 4% of videos (Table 2). Uploaders described 59% of tail-chasing videos as ‘Funny’, 26% as ‘Crazy’, 19% as ‘Cute’ and 15% as ‘Stupid’. Similarly, 46% of videos with comments from viewers were described as ‘Funny’ by the viewers, and 42% as ‘Cute’.

Viewers were 6.8 times more likely to describe perseverative dogs as ‘Funny’ (defined in Table 2) compared with more easily distracted dogs. Uploaders described dogs that tail-chased habitually as ‘Stupid’ (defined in Table 2) 6.5 times more often than other dogs. Examples of uploader comments describing habitual chasing are as follows: “*Ya it's funny she does this all the time:)*”; *“… my puppy does this ALL THE TIME. I've never seen a dog chase its tail so much. Maybe he enjoys the dizzyness??”*; “*This is just 1/100th of the allotted time [my dog] spends chasing his tail every day*”; “*This is him on a normal day. Chasing His Tail, Then eats his food, Watches a little TV, Chase's his tail some more then eat…*”; and (audible, rather than written) “*It's amazing how long he'll do that for… he never stops… it's your favourite game; you take it everywhere with you*”.

In nine videos (2.3%), at least one comment offered clinical explanations for the behaviour or suggested that the dog should be checked by a veterinarian (three comments by uploaders, and seven videos had at least one such comment by viewers). However, none of the descriptions indicated that uploaders had posted their video on YouTube™ specifically to raise awareness of clinical aspects of tail-chasing.

### Comparisons of environmental context and tail morphology against breed-matched controls

Videos showing tail-chasing were approximately 6.5 times less likely to be outdoors than were breed- and age-matched control videos (8.8% of tail-chasing videos were outdoors versus 38.8% of controls; Odds +/− S.E = 0.15+/−1.25; DF = 317; P<0.001); and when indoors, tail-chasing videos were over three times more likely to show a television or computer switched on than were controls (32.1% of indoor tail-chasing videos showed one switched on versus 9.1% of controls; Odds +/− S.E. = 3.35+/−1.34; DF = 106; *P*<0.001).

Control and tail-chasing videos showed no significant differences in tail morphology, such as length, docking, or hair-type (initial analyses had suggested that tails were longer in tail-chasing than control videos [26], but this relationship proved not to be robust when other significant variables were included in the final statistical models).

## Discussion

### Descriptions of tail-chasing characteristics, context and human responses to it

The results here reveal new clinically relevant information that has been difficult to discover previously. Approximately one third of the dogs with complete data tail-chased habitually or appeared perseverative, and were significantly more likely than other tail-chasers to be described as ‘Stupid’ or ‘Funny’, respectively. Comments suggesting clinical explanations for habitual, perseverative tail-chasing were only seen on 2.3% of videos, so it seems that public awareness must indeed be very low. Regardless of clinical signs, about one quarter (25.1%) of tail-chasing videos were classified as Comedy or Entertainment, laughter was recorded in over half (55%) of videos, and encouragement in 43%; and almost half of viewer comments described the videos as ‘funny’ or ‘cute’. The vast and ever growing numbers of viewings that these and similar videos receive on YouTube™ will likely reinforce these perceptions, normalising tail-chasing behaviour yet further [18].

The findings therefore indicate a gulf between public perception and indicators of poor welfare in tail-chasing dogs. This implies that many pathological tail-chasers may go untreated, and the behaviour is widely assumed to be normal and amusing regardless of its persistence. These results are perhaps not surprising considering that some owners also incorrectly perceive the – arguably less ambiguous – separation-related behaviours in their dogs (barking, whining, howling, scratching the door, destructive behaviour and inappropriate elimination) to indicate neutral or even positive welfare [27]. Similarly, owners can describe frequent signs of breathing difficulties in their brachycephalic (short-muzzle) dogs, but most later report that this not a ‘breathing problem’, being normal for the breed [28]. It appears that, although dogs seem readily to understand aspects of human behaviour [29], [30], humans do not necessarily interpret all important aspects of canine behaviour accurately.

Results in Table 3 show that problematic tail-chasing as a proportion of all the tail-chasing videos per breed group was prevalent in Bull Terrier breeds, consistent with clinical literature [4], [9], [12], but it was also widely distributed across other breed groups, including Toy and other groups little represented in studies to date. The prevalences here should not be taken as absolute values, because some breeds may be owned by a more technologically active demographic than others, and might thus be over represented on YouTube™. Also, if owners of breeds known to tail-chase compulsively are more aware of the clinical implications of this behaviour than other owners, they may be reluctant to post videos of it (e.g. being embarrassed or saddened by it), so those breeds could be under-represented. Nevertheless, the results indicate the degrees to which tail-chasing videos show problematic signs in the different breed groups and suggest that it would be worthwhile investigating whether there are hitherto unrecognized clinical implications of tail-chasing across diverse breeds. Possibly behavioural anomalies in small or toy dogs may be less likely to be referred for veterinary attention than in larger, heavier breeds, whose behaviour may be more disruptive and obviously problematic to the owners. A previous survey indicated that owners of smaller dogs may also be less attentive to their dogs’ behaviour and training in general [31].

In 17% of videos play behaviours were interspersed with tail-chasing; playing was less likely in perseverative dogs, but more likely in puppies than adult dogs. This is consistent with tail-chasing sometimes forming part of play, especially in puppies [4]. In these cases, as long as dogs infrequently chase their tails, owners need not necessarily be concerned about their dog's tail-chasing because play is often (but not always) an indicator of positive welfare [32]. A caveat is that even play can be a response to stress, lack of exercise or under-stimulation (a ‘do-it-yourself enrichment’, c.f. [33]), so owners should assess the context of the behaviour in case the trigger could be a negative one.

Encouragement of tail-chasing was recorded in 43% of videos, and laughter, which could also inadvertently be reinforcing for dogs, was heard in 55% of videos. The true prevalence of encouragement and laughter, will depend on how frequently people manipulate the dog for the film (e.g. attaching objects to the tail), play up to the camera, or deliberately remain quiet or offscreen during filming. Some encouragement seen on YouTube™ may have directly distressed the dogs: in almost 2% of videos, humans ‘growled’ at dogs, and almost 20% of people physically manipulated the tail (Table 2), often appearing to pull or pinch it with considerable force. In any case, whether reinforcement is through negative or positive means, it should be minimized to prevent tail-chasing from becoming compulsive. Equally, frequent tail-chasing must not be punished or prevented without addressing its cause, as this can increase stress and poor welfare in the affected dog, e.g. [34].

### Comparisons of environmental context and tail morphology in breed-matched controls

Compared with breed- and age-matched controls, tail-chasing videos were approximately 6.5 times less likely to be outdoors, and – when indoors – televisions or computers (but not radios or music players) were more frequently switched on. The breed- and age-matching was intended to control for some breeds being kept indoors to a greater extent than others. However, the environmental differences could still be Type I errors (falsely significant) if, for example, tail-chasing were one of the few canine behaviours that people tend to record indoors while watching television, rather than it being performed more in that situation *per se*. Some control videos were by nature likely to be filmed outdoors, such as dogs exercising or interacting with other dogs, but others showed more typically indoor activities, such as eating, dreaming, or interacting with other pets, so further research will be necessary to confirm the environmental contexts of tail-chasing.

Nevertheless, the observed environmental differences are consistent with tail-chasing being triggered by a lack of exercise, under-stimulation, and/or insufficient attention from humans [4], [7], [9], [11]. If so, the behaviour might indeed predominantly occur when dogs are indoors while humans are engaged in the sedate, non-interactive pastimes of television and computer use. Lack of exercise, stimulation and attention as triggers for tail-chasing have apparently not yet been tested empirically. If tail-chasing genuinely is associated with insufficient exercise, this would also be consistent with tail-chasing dogs having raised cholesterol levels, as found by Yalcin et al. [25].

The usual treatment for compulsive tail-chasing is drug therapy combined with behavioural therapy, such as increased owner attention and walks; the drugs may treat the clinical signs but behavioural change addresses the cause of the problem. However, owner compliance with behavioural recommendations is often poor, e.g. [7], and in general many dogs are walked very seldom (e.g. fewer than half of Australian owners surveyed walked their dogs at all [35], and 70% of dogs with acral lick dermatitis were never walked [36]). The finding that tail-chasing on YouTube™ appears to occur predominantly indoors with screens switched on might therefore reinforce the importance of exercise and stimulation for dogs.

Tail morphology and docking showed no significant differences between tail-chasing and control videos. A previous small-scale study [37] found neuromas in the docked tails of dogs showing ‘tail-directed behaviour’, so neuromas should be considered as a potential cause of tail-chasing in docked dogs, but no such association was found here (indeed the non-significant trend was in the opposite direction). A study focussing on breeds with frequently docked tails will be necessary to investigate whether a significant association exists.

### Conclusions

In summary, YouTube™ has offered the first large, study population of dogs chasing their tails in non-clinical contexts. Approximately one third of the dogs showed signs of clinical relevance, but this was rarely recognised openly by uploaders or viewers; indeed, dogs showing problematic tail-chasing were more likely than other dogs to be described as ‘Stupid’ or ‘Funny’. In 43% of videos tail-chasing was actively encouraged, which could risk reinforcing the behaviour excessively, and in some cases it included rough handling or goading the dog. The study also reveals that diverse dog breeds chase their tails on YouTube™, and that this seems predominantly to occur indoors when televisions or computers are switched on.

Future research could record more detail about the clinical signs: for example, details of tail-mouthing behaviour could indicate tail or hindquarter discomfort, and persistently chasing in one direction could help diagnose compulsivity [12]. It will also be necessary to determine what really triggers tail-chasing, to obtain meaningful prevalences of pathological and non-pathological tail-chasing, and to identify the most reliable indicators of whether the behaviour is of welfare concern. in the meantime, awareness of the clinical implications of frequent tail-chasing should be increased in the public domain if the associated canine welfare problems are to be addressed.

## Supporting Information

Table S1 **Condensed descriptions of all the data collected concerning YouTube™ videos of dogs chasing their tails.** * indicates that the data were also collected for breed-matched control videos.
(DOC)

Table S2 **The detailed description of criteria for scoring the presence or absence of particular characteristics in YouTube™ videos of dogs chasing their tails.** This includes a subset of the behavioural ethogram used to score the dog behaviour throughout the study. This summary was sent to the animal behaviour expert (OHB) who scored the 40 videos to allow inter-observer reliability to be tested.
(DOC)

Table S3 **Intra- and inter-observer reliability for selected variables describing dogs chasing their tails on YouTube™.** For each variable, the raw percentage agreement (%), the prevalence index (P.I.) and the κ value (for categorical variables) or W value (for ordinal variables) is shown. * indicates that the κ value fell below the clinically acceptable threshold of 0.4 (e.g. Sim & Wright, 2005), so the variable should be discarded from further analysis. ¥ indicates that the variable is ordinal, rather than categorical.
(DOC)
