# Supplementary material for: A Vicious Cycle: A Cross-Sectional Study of Canine Tail-Chasing and Human Responses to It, Using a Free Video-Sharing Website
Source: PLoS One. 2011 Nov 9;6(11):e26553. doi: 10.1371/journal.pone.0026553 (PMC3212522; doi:10.1371/journal.pone.0026553)
Supplement: Table S1 — Condensed descriptions of all the data collected concerning YouTube™ videos of dogs chasing their tails. * indicates that the data were also collected for breed-matched control videos. (DOC) [file pone.0026553.s001.doc]

**Table S1. Condensed descriptions of all the data collected concerning YouTube^TM^ videos of dogs chasing their tails.** * indicates that the data were also collected for breed-matched control videos.

| **Variable** | **Recording unit** | **Description (where necessary)** |
| --- | --- | --- |
| Video category | Free text | Video owners selected one of these from a list of categories determined by YouTube |
| Sex, Age and Country of Uploader | N/A | As reported on the Uploaders’ YouTube^TM^ homepages |
| Uploader descriptions | Free text | Adjectives describing how the Uploader perceived the tail-chasing (e.g. ‘funny’, ‘crazy’, ‘awesome’), or suggested motives for the dog to tail-chase (e.g. ‘bored’, ‘playing’); these were either written in the Uploader’s descriptions, as superimposed text on the video, or could be heard clearly during the video |
| Descriptive comments | Free text | As above, but using any comments written by the general public rather than the Uploader |
| Tail-chasing frequency | Habitual/ Periodic/ Rare | Uploader comments suggested that the dog tail-chased habitually (e.g. “All the time”, “Obsessed”)/ Uploader comments suggested that the dog tail-chased at least periodically (e.g. “From time to time”, “Regularly”, “[The dog] usually tail-chases when…”)/ Uploader comments suggested that the dog tail-chased rarely (e.g. “[The dog] rarely does this”, I “managed to catch” the dog tail-chasing) |
| Laughter | Yes/ No;  Male/ Female/ Both | Unambiguous laughter (voiced or ‘under the breath’) could be heard/ Sound quality was good but no laughter was heard; If laughter was heard, note was made if it was obviously male, female, or both sexes together |
| Encouragement | Verbal command/ Verbal Praise/ Physical intervention/ Physical praise/ Object attached/ None observed | Verbal commands e.g. “Get your tail”, “Get it”, or “Where’s your tail?”/ Praise, e.g. “Good dog” / A human was seen to touch, manipulate, squeeze, or pull tail / Dog was patted or stroked during or immediately after tail-chasing/ An object was attached to the tail/ No encouragement was observed |
| Difficult to distract | Yes/ No | The dog did not stop chasing for more than 5 s despite a potential distraction (e.g. the owner commanded the dog to do something other than tail-chase, a sudden noise, or the dog bumped into something hard enough to impede its progress)/ A potential distraction caused the dog to stop chasing for at least 5 s |
| Dog breed* | Breed name and breed group | Determined through the Uploader description, or if this was unavailable, it was estimated from the dogs’ appearances |
| Dog age* | Puppy/ Adult | <1 year of age, determined by Uploader description if available, or estimated from the appearance of the dog (e.g. small body size for the breed, large relative size of the head and feet, ‘fluffy’ fur texture, highly flexible movement etc)/ >1 year of age |
| Tail length* | Long / Medium/ Short/ None | Tail is at least half the length of the thoraco-lumbar spine (T-LS)/ Tail is between half and a quarter of the length of the T-LS / Tail is less than a quarter of the length of the T-LS, but wagging would be easily visible/ Tail is absent or too short to visibly move independently of the T-LS |
| Tail hair* | Very long/ Long/ Medium/ Short/ Curly | Hair obscures the outline of the tail and moves freely as the dog moves/ Hair obscures the lower outline of the tail, and moves freely as the tail moves/ Hair breaks the outline of the tail but moves little/ Tail outline appears smooth/ Tail is covered with curly fur not long enough to move freely |
| Tail tip* | Yes/ No | Up to half of the distal portion of the tail is a different colour from the proximal half/ The distal half of the tail is the same colour as at least some of the proximal half |
| Tail shape* | Straight/ Curved/ Curled | Tail has no obvious curve/ Tail curves consistently upwards or to one side/ Tail curls, touching itself or the dog’s hindquarters when at rest |
| Tail docked* | Yes/ No | Tail is noticeably shorter and ends more abruptly than the normal tail for the breed / Tail appears to taper towards the end and is a normal length for the breed |
| Barking | Yes/ No | At least one loud, sharp bark could be heard during or within 5 s of tail-chasing/ This was not heard/ Sound was absent or obscured |
| Growling | Yes/ No | Low-pitched growling or snarling could be heard during or within 5 s of tail-chasing/ This was not heard |
| Whining | Yes/ No | At least one high pitched whine or whimper could be heard during or within 5 s of a tail-chasing bout/ This was not heard |
| Panting | Yes/ No | The dog could be seen or heard to pant repeatedly with the mouth open during or with 5 s of tail-chasing/ This was not heard or seen |
| Collision | Yes/ No | Dog collides with an object during or up to 30 s after tail-chasing/ Dog is not observed to collide with anything |
| Falling to the ground | Yes/ No | Hindquarters contact the ground in an uncontrolled or ‘dizzy’ manner during or up to 30 s after a tail-chasing bout/ Hindquarters remain off the ground, or contact the ground in a smooth, controlled manner as the dog sits down. |
| Wagging | Yes/ No | Dog rhythmically moves its tail laterally at least twice in each direction within 5 s of a chasing bout/ Tail remains stationary or moves irregularly between chasing bouts |
| Play behaviour | Yes/ No | Within 5 s of a chasing bout the dog exhibits a play bow (characteristic posture with the forelegs extended on the ground), object play (manipulation of a toy or other available object), social play (with human or conspecific), or locomotor play (e.g., bounding, rolling)/ No obvious play behaviours are observed |
| Mouths tail | Yes/ No | Dog is clearly seen to bite, lick or hold the tail in its mouth for at least 1 s/ Dog is not seen to contact the tail with its mouth for 1 s or more |
| Mouths hindquarters | Yes/ No | As above, but concerning the hind leg, hindquarters or ano-genital region |
| Hairloss or wounds | Free text | Visible hairloss or injury to tail or hindquarters |
| Video location* | Inside/ Outside/ Unknown | Apparent through the light quality, ambient sound, and the background |
| Television/Computer switched on* | Yes/ No | Seen or heard in the video, or was referred to in the uploader description/ no clear evidence of this |
| Radio/Music playing* | Yes/ No | Heard in the ambient sound or referred to in the uploader description/ no clear evidence of this |
