# Supplementary material for: A Vicious Cycle: A Cross-Sectional Study of Canine Tail-Chasing and Human Responses to It, Using a Free Video-Sharing Website
Source: PLoS One. 2011 Nov 9;6(11):e26553. doi: 10.1371/journal.pone.0026553 (PMC3212522; doi:10.1371/journal.pone.0026553)
Supplement: Table S2 — The detailed description of criteria for scoring the presence or absence of particular characteristics in YouTube™ videos of dogs chasing their tails. This includes a subset of the behavioural ethogram used to score the dog behaviour throughout the study. This summary was sent to the animal behaviour expert (OHB) who scored the 40 videos to allow inter-observer reliability to be tested. (DOC) [file pone.0026553.s002.doc]

**Table S2. The detailed description of criteria for scoring the presence or absence of particular characteristics in YouTube^TM^ videos of dogs chasing their tails.** This includes a subset of the behavioural ethogram used to score the dog behaviour throughout the study. This summary was sent to the animal behaviour expert (OHB) who scored the 40 videos to allow inter-observer reliability to be tested.

***Ethogram for tail-chasing video inter-observer reliability***

*For all variables apart from ‘Ongoingness’ (tail-chasing frequency), most information is gained from watching the videos themselves, but for certain variables, some information can come from the written comments of the person who uploaded the video, or audible dialogue heard during the video.*

*For Ongoingness, information does not come from watching the dog’s behaviour. Instead it comes from written and audible comments, and it can also come from the uploader’s other videos (the uploader’s name appears as a hyperlink below the video, but you don’t need to click this if you have sufficient information about ongoingness from the video page itself). Be careful to get the right dog if the uploader has videos of other dogs! Also bear in mind that if the dog tail-chases regularly, it may do it in other videos and yet the uploader might not consider it a significant feature of the video, so they can be easy to miss.*

*All data are binary (‘y’/’n’), except Ongoingness (0,1,2), as described below. The descriptions for the binary variables below indicate an answer of ‘y’ (meaning ‘yes, this occurs during the video’ or ‘yes, this describes this aspect of the video’), so if the video does not fit the description, the answer is ‘n’.*

*Missing values are permissible (coded as ‘xx’), but try to avoid them if you can. The variables can be hard to decide about at times. For variables where the ambient soundtrack is important, you will need to put missing values whenever the soundtrack is overlaid by music or is absent.*

Thank you.

| **Behavioural characteristic** | **Description** |
| --- | --- |
| Ongoingness? | 2 = ‘Habitual’, e.g. can see from the comments or can hear in the sound track that the dog chases daily, “all the time”, “a lot”, “spends hours” tail-chasing, or is “obsessed” |
|  | 1 = ‘Periodical’, e.g. can see from the comments or can hear in the sound track that the dog chases “from time to time”, “regularly”, “[the dog] usually tail-chases when…” OR can see from the Uploader’s other videos that the dog does this in other contexts or at different ages, i.e. it is shown tail-chasing on at least two different occasions. |
|  | 0 = ‘Rare’, e.g. can see from the comments or can hear in the sound track that the dog chases “rarely”, “occasionally”, that the videographer “managed to catch” the dog tail-chasing |
| Difficult to Distract? | The dog does not stop chasing for more than 5 s despite a potential distraction (e.g. the owner commanded the dog to do something other than tail-chase, a sudden noise, or a collision hard enough to impede the dog’s progress). If more than one distraction occurs, the dog appears difficult to distract – according to these aforementioned criteria – on at least half of the occasions. If no obvious distractions occur, this is a missing value. The comments should NOT be used to determine distractibility. |
| Play behaviours? | Within 5 s before or after a tail-chasing bout, the dog exhibits a play bow (characteristic posture with the forelegs extended on the ground), object play (manipulation of a toy or other available object), social play (with human or conspecific), or locomotor play (e.g., bounding, jumping, rolling). Rolling does not count if it results from the dog falling over but continuing to reach for its tail. |
| Falls over? | Hindquarters contact the ground in an uncontrolled or ‘dizzy’ manner during, or up to 30 s after, a tail-chasing bout. The dog sitting down in a controlled manner does not count. |
| Collision? | Dog collides with an object during or up to 30 s after tail-chasing. Even a soft object can count, as long as it is something a dog would avoid colliding with when walking normally. |
| TV or computer? | A television or computer appears to be in use: a screen, or part of it, may be clearly seen; it may be heard (e.g. the tapping of computer keys or dialogue with a pre-recorded sound quality to it, which is interspersed with longer pauses than would occur on radio); or emitted light from televisions can be seen if it illuminates the scene with sudden transitions between changeable coloured light. In addition, the comments can reveal that the TV/computer was on (and perhaps switched off or muted for the purposes of the video recording), e.g. “I was just watching X when the dog started tail-chasing”. |
| Radio or stereo? | A continuous ambient (rather than overlaid) soundtrack can be heard, e.g. if this is music, it will be continuous or broken by pauses of <5 seconds, and if it is dialogue, it will sound pre-recorded (rather than like actual people talking in the room), and will have no real pauses, unlike a television soundtrack. As above, the comments can reveal whether the radio or stereo is on. |
| Outside? | The scene is obviously outside rather than inside as indicated by the light quality, background, and any ambient sounds. The ground and walls may be man-made, e.g. concrete. |
| Laughter | Laughter can be heard. This may be overt or ‘under the breath’. It must not be part of a television or radio soundtrack. |
| Encouragement | Can include verbal or physical praise, verbal encouragement (e.g. “get your tail”), or physical manipulation of the tail, including attaching objects to the tail. In videos with no ambient soundtrack, a missing value should be recorded unless definite physical encouragement is seen, because verbal encouragement cannot be ruled out. |
| Whining? | The dog emits a relatively quiet, drawn-out, high-pitched vocalisation during or within 5 s of tail-chasing |
| Growling? | The dog emits a low-pitched rumbling vocalisation during or within 5 s of tail-chasing |
| Barking? | The dog emits a loud, sharp vocalisation during or within 5 s of tail-chasing |
| Panting? | The dog is seen or heard to pant repeatedly with the mouth open during or within 5 s of tail-chasing |
| Wagging? | Dog rhythmically moves its tail laterally at least twice in each direction within 5 s of a chasing bout, rather than the tail moving irregularly |
| Mouths tail? | Dog is clearly seen to bite, lick or hold its tail in its mouth for at least 1 s |
| Mouths hindquarters? | Dog is clearly seen to bite, lick or hold its hindquarters or hind leg in its mouth for at least 1 s |
