# Supplementary material for: A Vicious Cycle: A Cross-Sectional Study of Canine Tail-Chasing and Human Responses to It, Using a Free Video-Sharing Website
Source: PLoS One. 2011 Nov 9;6(11):e26553. doi: 10.1371/journal.pone.0026553 (PMC3212522; doi:10.1371/journal.pone.0026553)
Supplement: Table S3 — Intra- and inter-observer reliability for selected variables describing dogs chasing their tails on YouTube™. For each variable, the raw percentage agreement (%), the prevalence index (P.I.) and the κ value (for categorical variables) or W value (for ordinal variables) is shown. * indicates that the κ value fell below the clinically acceptable threshold of 0.4 (e.g. Sim & Wright, 2005), so the variable should be discarded from further analysis. ¥ indicates that the variable is ordinal, rather than categorical. (DOC) [file pone.0026553.s003.doc]

**Table S3. Intra- and inter-observer reliability for selected variables describing dogs chasing their tails on YouTube^TM^. For each variable, the raw percentage agreement (%), the prevalence index (P.I.) and the κ value (for categorical variables) or W value (for ordinal variables) is shown. * indicates that the κ value fell below the clinically acceptable threshold of 0.4 (e.g. Sim & Wright, 2005), so the variable should be discarded from further analysis. ¥ indicates that the variable is ordinal, rather than categorical.**

| **Variable** | **Intra-observer reliability** | | | **Inter-observer reliability** | | |
| --- | --- | --- | --- | --- | --- | --- |
|  | **%** | **P.I.** | **κ or W value** | **%** | **P.I.** | **κ or W value** |
| Barking | 97.2 | 0.75 | 0.87 | 94.4 | 0.77 | 0.77 |
| Collisions | 85.0 | 0.45 | 0.62 | 72.5 | 0.36 | 0.43 |
| Difficult to distract | 89.7 | 0.21 | 0.78 | 76.5 | 0.33 | 0.51 |
| Encouragement | 91.9 | 0.00 | 0.84 | 89.5 | 0.11 | 0.79 |
| Falling over | 92.5 | 0.43 | 0.94 | 82.5 | 0.40 | 0.61 |
| Growling | 88.9 | 0.56 | 0.68 | 88.9 | 0.65 | 0.68 |
| Human laughter | 97.2 | 0.25 | 0.94 | 94.4 | 0.26 | 0.88 |
| Mouthing hindquarters | 95.0 | 0.85 | 0.64 | 92.5 | 0.90 | 0.53 |
| Mouthing tail | 82.5 | 0.13 | 0.64 | 97.5 | 0.14 | 0.95 |
| Tail-chasing frequency^¥^ | 83.3 | N/A | 0.89 | 73.5 | N/A | 0.84 |
| Outside/inside | 100.0 | 0.75 | 1.00 | 100.0 | 0.84 | 1.00 |
| Panting | 73.0 | 0.24 | 0.43 | 70.3 | 0.37 | 0.39^*^ |
| Play behaviour | 90.0 | 0.45 | 0.75 | 82.5 | 0.56 | 0.57 |
| Television/computer use | 85.0 | 0.30 | 0.67 | 80.6 | 0.45 | 0.57 |
| Tail wagging | 75.0 | 0.30 | 0.45 | 72.5 | 0.36 | 0.43 |
